# Cross-sectional comparison of health care delivery and reimbursement between segregated and nonsegregated communities in Hungary

**DOI:** 10.3389/fpubh.2024.1152555

**Published:** 2024-01-24

**Authors:** Feras Kasabji, Ferenc Vincze, Kinga Lakatos, Anita Pálinkás, László Kőrösi, László Ulicska, Karolina Kósa, Róza Ádány, János Sándor

**Affiliations:** ^1^Department of Public Health and Epidemiology, Faculty of Medicine, University of Debrecen, Debrecen, Hungary; ^2^ELKH-DE Public Health Research Group, Department of Public Health and Epidemiology, Faculty of Medicine, University of Debrecen, Debrecen, Hungary; ^3^National Health Insurance Fund, Budapest, Hungary; ^4^Deputy State Secretariat for Social Inclusion, Ministry of Interior, Budapest, Hungary; ^5^Department of Behavioral Sciences, Faculty of Medicine, University of Debrecen, Debrecen, Hungary

**Keywords:** cross-sectional, segregation, inequality, healthcare, health reimbursement, general medical practitioner, Hungary

## Abstract

**Introduction:**

Spatially segregated, socio-economically deprived communities in Europe are at risk of being neglected in terms of health care. In Hungary, poor monitoring systems and poor knowledge on the health status of people in these segregated areas prevent the development of well-informed effective interventions for these vulnerable communities.

**Aims:**

We used data available from National Health Insurance Fund Management to better describe health care performance in segregated communities and to develop more robust monitoring systems.

**Methods:**

A cross-sectional study using 2020 health care data was conducted on each general medical practice (GMP) in Hungary providing care to both segregated and nonsegregated (complementary) adult patients. Segregated areas were mapped and ascertained by a governmental decree that defines them as within settlement clusters of adults with low level of education and income. Age, sex, and eligibility for exemption certificate standardized indicators for health care delivery, reimbursement, and premature mortality were computed for segregated and nonsegregated groups of adults and aggregated at the country level. The ratio of segregation and nonsegregation specific indicators (relative risk, RR) was computed with the corresponding 95% confidence intervals (95% CI).

**Results:**

Broad variations between GMPs were detected for each indicator. Segregated groups had a significantly higher rate of health care service use than complementary groups (RR = 1.22, 95% CI: 1.219;1.223) while suffering from significantly reduced health care reimbursement (RR = 0.940, 95% CI: 0.929;0.951). The risk of premature mortality was significantly higher among segregated patients (RR = 1.184, 95% CI: 1.087;1.289). Altogether, living in a segregated area led to an increase in visits to health care services by 18.1% with 6.6% less health spending.

**Conclusion:**

Adults living in segregated areas use health care services more frequently than those living in nonsegregated areas; however, the amount of health care reimbursement they receive is significantly lower, suggesting lower quality of care. The health status of segregated adults is remarkably lower, as evidenced by their higher premature mortality rate. These findings demonstrate the need for intervention in this vulnerable group. Because our study reveals serious variation across GMPs, segregation-specific monitoring is necessary to support programs sensitive to local issues and establish necessary benchmarks.

## 1 Introduction

Segregation, whether residential, racial, or otherwise, is a recognized risk factor for ill health and inequity. Various studies have explored segregation and its impact on health (1–7) and found pronounced inequality in health care outcomes between segregated and nonsegregated areas, including but not limited to overall infant and adult mortality, high-risk pregnancies, and exposure to communicable diseases (7, 8). Reasons for these disparities range from environmental factors, such as sanitation facilities and pollution, to lifestyle factors, such as poor housing situations, crowding, and habits such as poor diets, smoking, and low physical activity (9, 10).

The relationship between segregation and health is well documented. However, the specific association of segregation with health care delivery and reimbursement policies is still understudied. A prominent example of this is the Roma people in Europe, who constitute the largest ethnic minority in Europe (11, 12) and in Hungary (13), where they make up 94% of inhabitants in segregated areas (14). Data regarding their health are limited. However, it is estimated that Roma have a 10-year shorter life expectancy (15) and are at higher risk of coronary and chronic diseases (16, 17). It makes them among the most relevant health equity concerns in Europe and prompts many studies to investigate segregated Roma access to and use/misuse of health care services (18–22). However, these studies have fallen short of explaining the determinants of the observed health losses. This shortfall has contributed to the failure of governments to implement effective equity-targeted policies, as evidenced by the current dissimilarity in health between Roma and non-Roma populations (23).

A major constraint of any Roma-focused study is the inability to accurately identify Roma ethnicities in local demographics or estimate their health statistics, mainly due to ethical issues and the unavailability of a clear-cut method to do so (24–26). These limitations generate unguided financial policies and governance, leading to considerable inequality in health delivery and outcomes both geographically and among population groups (27, 28). This situation gives rise to the need for more robust monitoring and intervention programs, both of which require a clearer picture of the variability in health care delivery and reimbursement.

Moreover, even though Roma are overrepresented in segregated communities in Hungary, the non-Roma population of the segregates lives in equal socioeconomic deprivation, and interventions should therefore be sensitive to this situation and the local environment and aim to help the vulnerable population regardless of ethnic background.

As a result, current inclusion policies shift the focus from Roma people to segregated communities regardless of ethnicity, to avoid any ethical concerns surrounding the identification of different ethnic groups as well as monitoring their health status, in accordance with the Hungarian national social inclusion strategies (29).

In Hungary, the National Institute of Health Insurance Fund Management (NIHIFM) is the organization operating the country’s official health monitoring system. Every month, NIHIFM evaluates all general medical practices (GMPs) by a limited set of performance indicators (30, 31), which then affect GMP financing through a pay-for-performance system. More data on primary health care operations are collected but not utilized by the NIHIFM, leaving an untapped reservoir of data that could be useful for research.

Using the protected data within the NIHIFM system, aggregated indicators can be produced for segregated areas where most inhabitants are Roma and other vulnerable groups that are cared for by identifiable institutions, achieved by linking the geographical location of segregation with the health-insured population living there. These aggregated statistics can then be used to investigate the most vulnerable subgroup of Roma by evaluating geographical inequality (32, 33), thus bypassing legal and ethical limitations. The governmental decree’s definition of segregation utilizes census data, focusing on income and education measures rather than Roma ethnicity, which enables the study of segregation related issues and leads to conclusions indirectly related to Roma.

The purpose of our study was (1) to use the available but untapped NIHIFM data as a means to describe patient inequality in health delivery and reimbursement between segregated communities (where the most vulnerable populations with extremely high Roma representation reside) and nearby nonsegregated areas; (2) to outline the variability of this inequality across different Hungarian GMPs that serve both segregated and nonsegregated areas; and (3) to aid in the conceptualization and implementation of a new equality-oriented monitoring system.

## 2 Methods

### 2.1 Setting

The study utilized person-level health records, and evaluated GMP-level-aggregated indicators. All Hungarian GMPs (*N* = 4,359) who delivered care for adults were investigated. Each of them were contracted with the NIHIFM (the only health insurance institute in Hungary). The NIHIFM provided data on 2020 for secondary analysis on the health care use, reimbursement, and health status of adults belonging to the GMPs.

### 2.2 Design

A cross-sectional study of Hungarian GMPs providing care to segregated adults was performed. Segregated areas (SAs) were mapped and ascertained by applying the classification of a governmental decree that defines them as within settlement clusters of adults between the ages of 18 and 59 with a high proportion with at most primary-level education and no active income. Accordingly, the NIHIFM classified each household as either an SA or nonsegregated area [complementary area (CA)], which were mutually exclusive. Using addresses, any adult of at least 18 years of age could therefore be defined as living in an SA or *CA.* GMPs without patients living in an SA were excluded from the analysis.

#### 2.2.1 Outcome indicators

##### 2.2.1.1 Health care delivery

Health care delivery rates for multiple services were calculated as the number of patients who used the health service per patient belonging to a GMP for the previous 12 months. The delivery indicators included (1) the number of general practitioner visits, (2) outpatient service use without computed tomography or magnetic resonance imaging (CT/MRI) services, (3) CT/MRI services, and (4) hospitalization.

##### 2.2.1.2 Health care reimbursement

*Per capita* reimbursement was calculated for these services as health insurance spending (in Euros) per patient belonging to the general medical practice for the previous 12 months. With the addition of medication costs, and since the NIHIFM finances GMPs *per capita* irrespective of the number of patient visits, this measure was not included among the reimbursement indicators, as it does not influence variability in GMP average *per capita* financing.

##### 2.2.1.3 Premature mortality

All-cause premature mortality was defined as all deaths of adults below 65 years of age who had not changed their GMP in the past 5 years. This restriction was applied to exclude those who died in the care of a new GMP who did not manage their health prior to their death.

### 2.3 Statistical analysis

Standardized performance measures were calculated using the national average as a reference. Indicators for SAs and CAs were indirectly standardized by age (applied age groups: 18–24, 25–29, 30–34, 35–39, 40–44, 45–49, 50–54, 55–59, 60–64, 65–69, 70–74, 75–79, and 80 and above), sex and, eligibility for an exemption certificate. Exemption certificates are issued by the local municipalities to patients with disadvantageous socioeconomic status and chronic diseases (if recommended by GPs) and ensure free of charge access to medicines and medical devices. If GMPs provided care to more than one settlement, the observed and expected values were summarized to obtain GMP specific SA and CA measures. Dividing GMP-level observed values over expected values resulted in standardized risk ratios for SAs (SRsa) and CAs (SRca) for each indicator and GMP (32, 34–36). These GMP-level data were aggregated further to obtain country level standardized measures for SAs and CAs.

Relative performance in SAs was described by the risk ratio (RR), which was calculated by the SRsa/SRca ratio for each GMP and for the whole country along with 95% confidence intervals (95% CIs). Impact measures such as excess number of cases in the SAs, percentage of risk attributable to segregation in the population of SAs (attributable risk), and percentage of risk attributable to segregation in the population of the entire country (population attributable risk) were computed using nationally and locally adjusted standardized ratios.

## 3 Results

**Figure 1:**
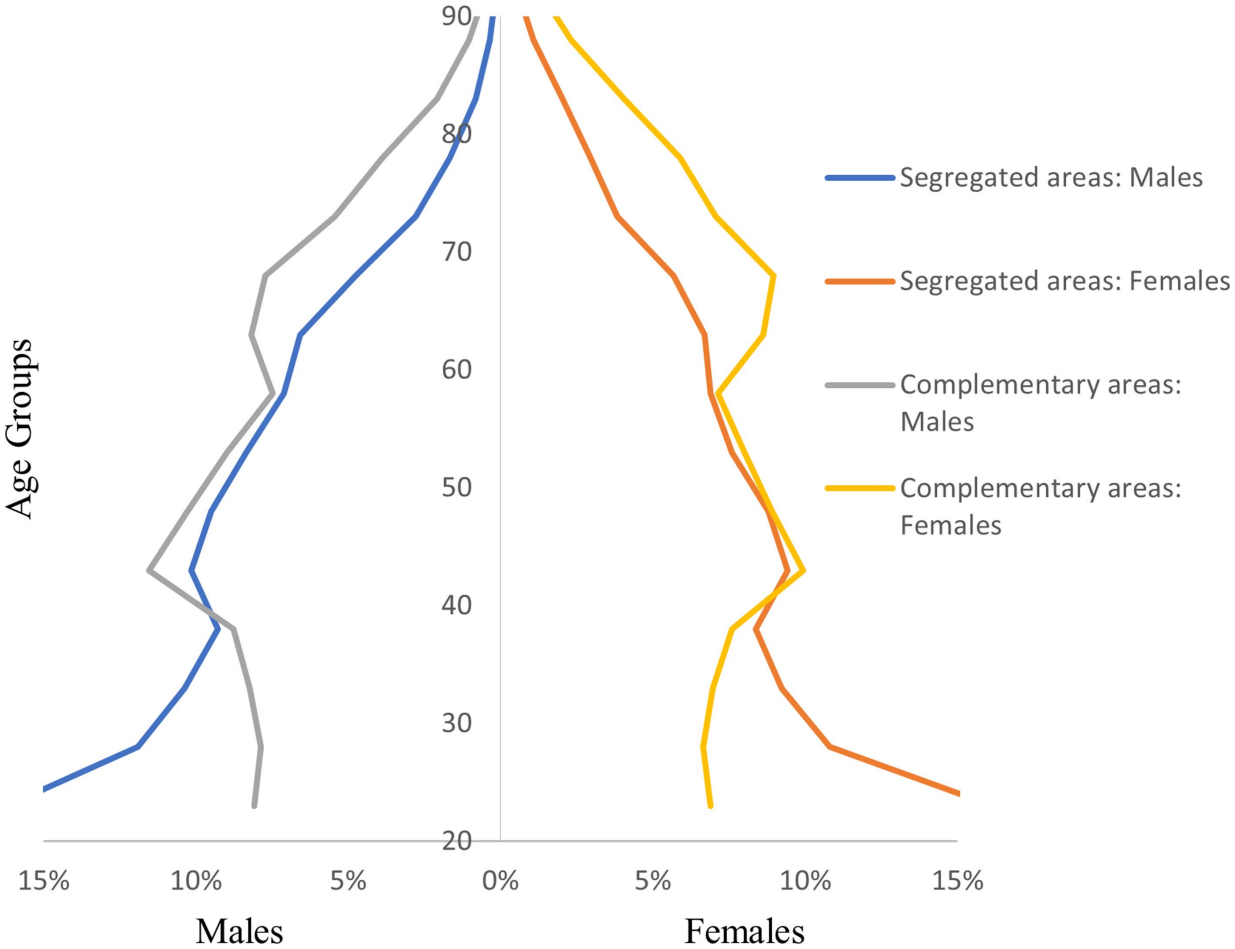
Demographic structure of the segregated and complementary populations.

The studied population consisted of 7,385,641 adults (3,456,560 men and 3,929,081 women), with 2,071 identified segregated areas hosting 283,876 adults (139,507 men; 144,369 women). Demographic structure varied widely between the SA and CA populations (Figure 1). The mean age was notably lower among those living in SAs (total: 43.3 years; men: 42.2 years; women: 44.4 years) than among those living in CAs (total: 50.4 years; men: 48.5 years; women: 52.1 years). The older adult dependency ratio (the proportion of older adult individuals aged 65 or above among individuals aged 15 and above) was remarkably lower in SAs (15.4%) than in CAs (33.7%).

**Figure 2:**
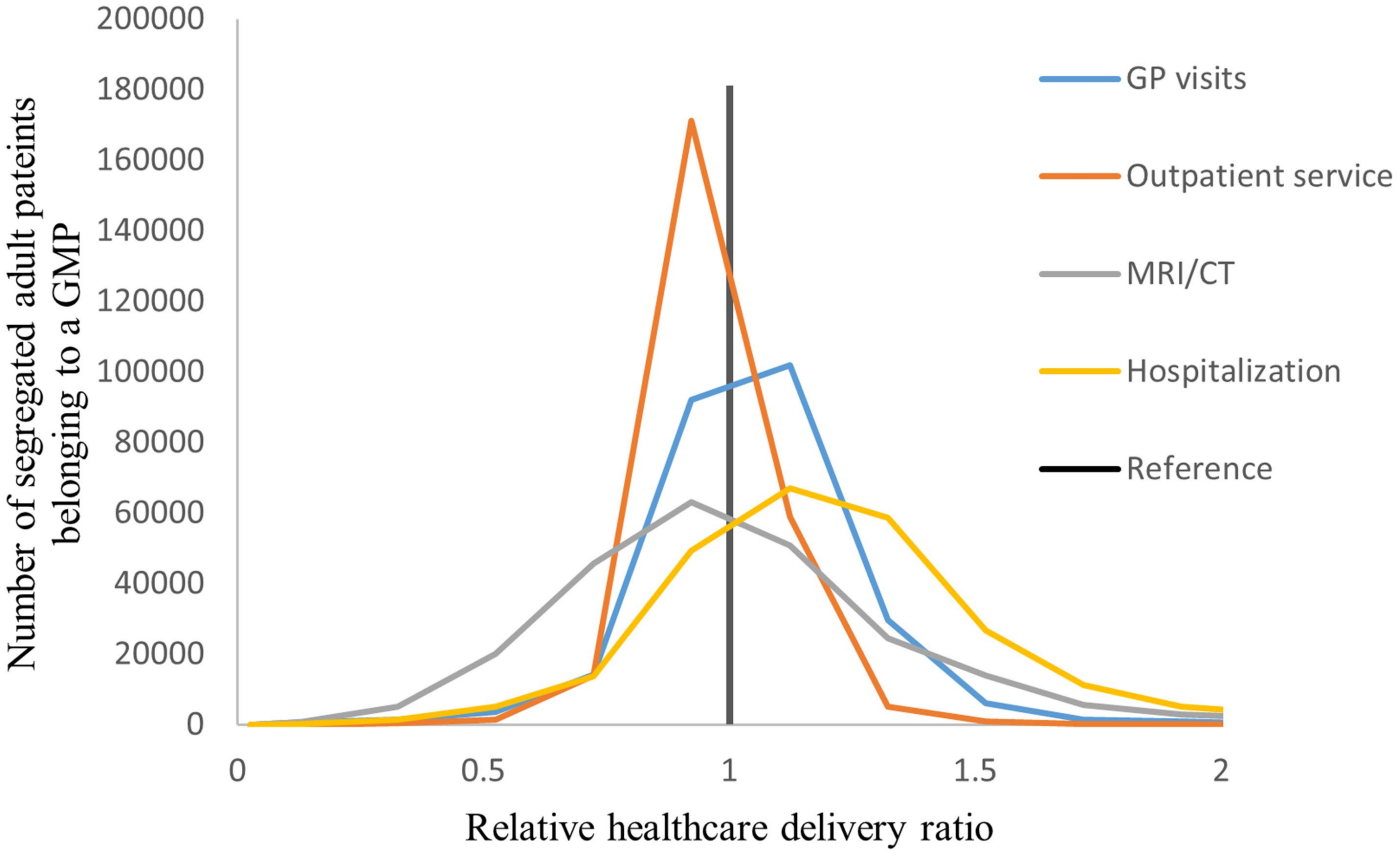
Distribution of segregated adult patients according to the relative health care delivery ratio among Hungarian GMPs.

**Figure 3:**
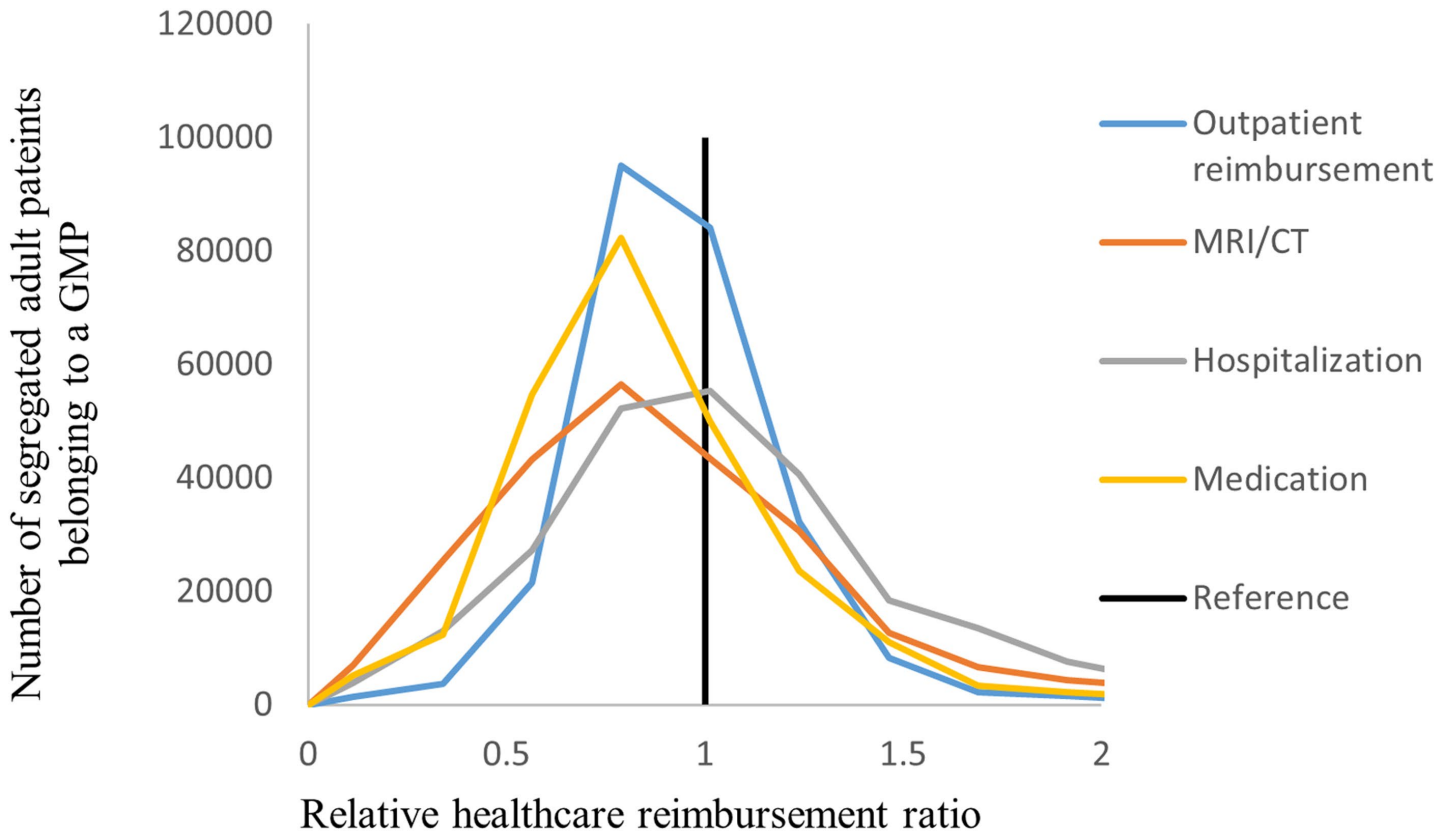
Distribution of segregated adult patients according to the relative health care reimbursement ratio among Hungarian GMPs.

**Figure 4:**
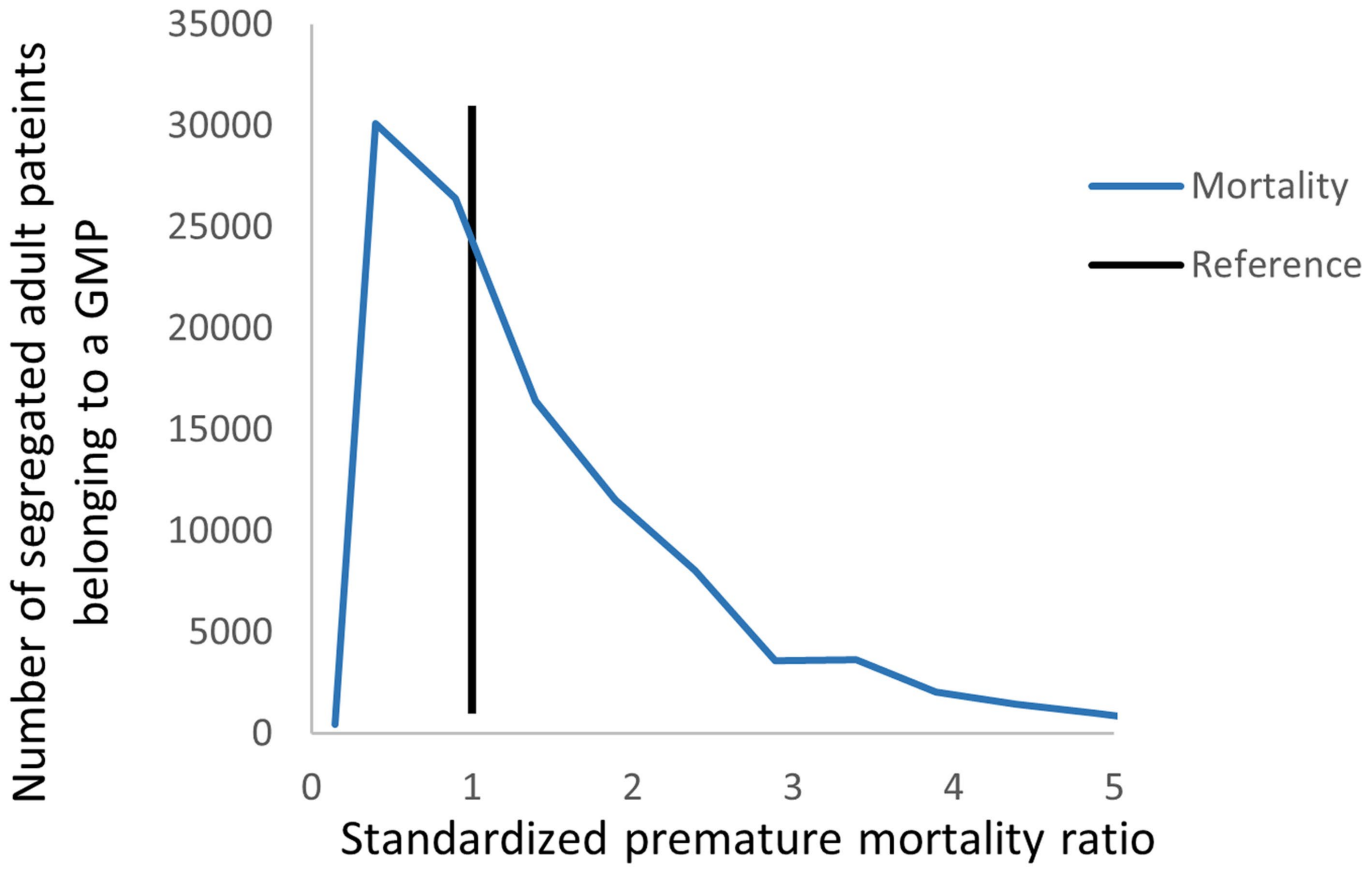
Distribution of segregated adult patients according to the relative premature mortality ratio among Hungarian GMPs.

The distribution of the number of segregated patients belonging to a GMP was highly varied across the standardized health care delivery, health care reimbursement, and premature mortality indicators (Figures 2–4). The highest level of heterogeneity of both episode and reimbursement indicators for relative GMP performance was observed in imaging examinations. Outpatient service use showed the highest variability.

### 3.1 Health care delivery

According to the standardized relative indicators aggregated for the whole country (Table 1) segregated groups had a significantly higher rate of health care services use than their complementary counterparts (RR = 1.222, 95% CI: 1.220;1.223). Specifically, for individual services provided to segregated patients, the number of GP visits per person per year (RR = 1.251, 95% CI: 1.249;1.253) and the number of hospital admissions per year exceeded those for complementary patients (RR = 1.250, 95% CI: 1.237;1.264). on the other hand, segregated patients experienced a significantly reduced number of treatments per year in outpatient service centers (RR = 0.948, 95% CI: 0.943;0.953) and imaging examinations (RR = 0.935, 95% CI: 0.920;0.950) compared to their counterparts.

**Table 1:** Relative health care delivery, reimbursement, and all-cause premature mortality among Hungarian adults provided by general medical practices situated in a segregated or complementary area.

Indicators	Total	Segregated colonies		Complementary areas		Relative performance in segregated colonies [95%CI^#^]
	*N*	*N*	Standardized performance* [95%CI^#^]	*N*	Standardized* performance [95%CI^#^]	
Healthcare delivery (episodes)						
GP visits	47,754,032	1,993,344	1.238 [1.237;1.240]	45,760,688	0.990 [0.989;0.990]	1.251 [1.249;1.253]
Use of outpatient services without CT/MRI	4,522,976	150,414	0.951 [0.946;0.956]	4,372,562	1.003 [1.002;1.004]	0.948 [0.943;0.953]
Use of CT/MRI	493,566	15,078	0.940 [0.925;0.955]	478,488	1.005 [1.002;1.008]	0.935 [0.920;0.950]
Use of hospital service	836,818	35,527	1.241 [1.228;1.254]	801,291	0.992 [0.990;0.994]	1.250 [1.237;1.264]
Total	53,607,392	2,194,363	1.211 [1.209;1.212]	51,413,029	0.991 [0.990;0.991]	1.222 [1.220;1.223]
Healthcare reimbursement (Euro *per capita*)						
Use of outpatient services without CT/MRI	42.67	35.31	0.885 [0.856;0.915]	42.94	1.008 [1.002;1.014]	0.878 [0.848;0.908]
Use of CT/MRI	8.57	6.34	0.823 [0.760;0.890]	8.65	1.009 [0.996;1.022]	0.815 [0.752;0.883]
Hospital service	124.63	117.36	1.062 [1.043;1.082]	124.91	0.999 [0.996;1.003]	1.063 [1.043;1.083]
Use of hospital service	147.18	130.01	0.871 [0.856;0.887]	147.82	1.003 [0.999;1.006]	0.869 [0.854;0.884]
Total	323.05	289.02	0.940 [0.929;0.952]	324.33	1.002 [1.000;1.004]	0.938 [0.927;0.950]
All cause premature mortality						
All-cause premature mortality	23,453	1,208	1.087 [1.027;1.150]	22,245	0.996 [0.983;1.009]	1.092 [1.030;1.157]

^*^Age, sex, and eligibility for exemption certificate standardized. ^#^95% confidence interval.

Regarding attributable risk (Table 2), patients living in an SA were associated with an increase in visits to health care services by 18.1% (95% CI: 18.0;18.2), with a population-attributable risk of 0.742% when comparing with CAs. Considering the number of episodes, the number of GP visits showed broad inequality, with an estimation of 400,024 (95% CI: 397,811;402,234) excess visits made per year. Hospital service use also had an excess of 7,116 (95% CI: 6,819;7,410), a 20% increase compared to residence in a *CA.* Meanwhile, the number of outpatient services used and CT/MRI examinations were lower by 8,241 (95% CI: 9,045;7,441) and 1,046 (95% CI: 7,911;306), respectively.

**Table 2:** Impact of segregation among adults living in segregated areas (number of excess cases and attributable risk) and in the whole adult population of Hungary (population attributable risk).

Indicators	Number of excess cases [95%CI^#^]	Attributable risk [95%CI^#^]	Population attributable risk
Healthcare delivery (episodes)			
GP visits	400,024 [397,811;402,234]	20.1% [20.0%;20.2%]	0.838%
Use of outpatient services without CT/MRI	−8,241 [−9,045;−7,441]	−5.5% [−6.0%;−4.9%]	−0.182%
Use of CT/MRI	−1,046 [−1,306;−791]	−6.9% [−8.7%;−5.2%]	−0.212%
Use of hospital service	7,116 [6,819;7,410]	20.0% [19.2%;20.9%]	0.850%
Total	397,921 [395,543;400,297]	18.1% [18.0%;18.2%]	0.742%
Healthcare reimbursement (Euro *per capita*)			
Use of outpatient services without CT/MRI	−477 [−609;−348]	−14.0% [−17.8%;−10.2%]	−0.418%
Use of CT/MRI	−139 [−201;−81]	−22.7% [−32.8%;−13.3%]	−0.607%
Hospital service	674 [476;869]	5.9% [4.2%;7.7%]	0.203%
Medication	−1,899 [−2,154;−1,648]	−15.1% [−17.1%;−13.1%]	−0.483%
Total	−1,838 [−2,190;−1,491]	−6.6% [−7.8%;−5.3%]	−0.213%

### 3.2 Health care reimbursement

Health care reimbursement also had significant dissimilarities depending on whether patients lived in SAs or CAs (Table 1). Total health services reimbursement for a GMP was significantly reduced for SA patients (RR = 0.938, 95% CI: 0.927;0.950). Specifically, GMPs exhibited significantly lower reimbursement per year for outpatient services to SA patients (RR = 0.878, 95% CI: 0.848;0.908), MRI/CT examinations (RR = 0.815, 95% CI: 0.752;0.883), and medications (RR = 0.869, 95% CI: 0.854;0.884). Hospitalization reimbursement, on the other hand, showed higher spending on segregated groups (RR = 1.063, 95% CI: 1.043;1.083).

Providing care to segregated patients lowered health care reimbursement by 6.6% (95% CI: 7.8;5.3) (Table 2) when compared to complementary groups, with a population-attributable risk of −0.213%. This finding is predominantly attributed to medication-related NIHIFM reimbursement, where the *per capita* yearly cost was lower for SA patients by 1,899 EUROs (95% CI: 2,154;1,648) compared to CA patients.

### 3.3 All-cause premature mortality

The age-and sex-standardized premature mortality among the SA population was significantly higher than that in the CA population (RR = 1.092, 95% CI: 1.030;1.157) (Table 1). The estimated 101.544 (95% CI: 37.355;162.213) excess cases in the SA population corresponded to 8.406% (95% CI: 3.092;13.428). The population level impact was estimated to be 0.433%.

## 4 Discussion

### 4.1 Main findings

Our study reveals variation and statistically significant dissimilarity in health care use, reimbursement, and premature mortality across Hungarian GMPs, pointing to causes of the existing health gap between segregated people and their counterparts living in complementary areas.

A characteristic health care use pattern of adults living in segregated areas was identified. People living in SAs use health care services more frequently than those living in CAs; however, the amount of health care reimbursement paid for their care is significantly lower, suggesting lower quality of care.

In the case of primary care, crude indicators show that segregated groups had a higher rate of GP appointments, which corresponds with other studies from Europe (37, 38). And publications on the poorer health status of residents of segregations (32, 35, 36).

However, the services of outpatient and imaging centers were notably less utilized by segregated groups, which correlates with a significant reduction in GMPs’ reimbursement compared to complementary groups. This difference, coupled with the fact that people living in SAs have poorer health status and a likely greater need for outpatient services, implies the underutilization of these services by segregated groups.

This finding helps explain the increase in hospital service utilization in SA groups paralleled with an increase in reimbursement; since outpatient services (including advanced and specialized tests and treatments) are required to diagnose, reverse, or halt the progression of chronic diseases, which can otherwise go undetected until a more serious prognosis requires hospitalization. Moreover, this discrepancy could also be due to the poorer health and greater needs of people living in SAs. Numerous studies have reported increased hospital admissions, avoidable and otherwise, in segregated minorities (32, 39, 40).

The health status of adults living in segregation was significantly poorer than that of adults living in complementary areas, as evidenced by their higher premature mortality rate, corroborating other studies showing the same conclusion (17, 34, 35). Their poorer health may be an outcome of their lower socioeconomic status and unhealthy lifestyles (10, 41) combined with the provision of lower-quality health care (30).

### 4.2 Strengths and limitations

The major strength of our study was the inclusion of all Hungarian adults living in SAs or in CAs. Because the Hungarian GMPs are required to contract with NIHIFM, detailed reimbursement and health data were available for each subject. Therefore, there was no selection bias in our study. Further, the proper quality of data was ensured by the standard NIHIFM protocols for data collection (42).

The present study results reflect estimations of health care use and mortality of Roma living in SAs, since Roma constitute 94% of the inhabitants of these areas (14). It is presumable that the Roma and non-Roma adults in SAs follow similar lifestyle and have similar health status. Consequently, the SA specific indicators can be considered as proxy estimations for those Roma who live in segregated areas.

Unfortunately, the health care use indicators of our study were not adjusted for the health needs and health status of the investigated populations, to which the observed differences in health care use could be partially attributed. The higher premature mortality shows that the health status is worse in SAs, consequently leading to more intensive use of health care services in these areas. In this context, the higher frequency of GP visits and hospital admissions can be considered an adequate response to the higher health care needs, while the lower utilization of outpatient services in SAs is inconsistent, suggesting a potential malfunction in the system, where fewer outpatient services were provided than necessary. Our results do not identify the mechanisms behind the observed inequalities, calling for more detailed pathway analyses to propose interventions that could help lessen the present inequality.

Data available for our analysis were from 2020. That year was seriously affected by COVID-19. The first case in Hungary was detected on 04/03/2020 (43). The consecutive epidemiologic measures profoundly impacted health care operation (44). Consequently, our results predominantly reflect health care inequality during the COVID-19 epidemic. However, the compared groups of SAs and CAs shared the same settlements, geographical access to health care, and GP who was ultimately responsible for the gate-keeping of treatment pathways. Therefore, any differences between SAs and CAs in health care use or reimbursement should be minimized. Nevertheless, vaccination coverage was lower in SAs, suggesting that the COVID-19 epidemic contributed to some of the observed health care use and reimbursement inequalities (45). Altogether, the inequality pattern observed in our study should be further investigated in years not affected by the epidemic to substantiate the findings of our investigation.

### 4.3 Implications

Our findings indicate a significant association between segregation and severe health care issues, as demonstrated by country level aggregated relative risk measures. Notably, these problems largely stem from the local setting as evidenced by the varying levels of inequality at the local GMP-level. Some GMPs exhibit pronounced local inequalities, while others show no such disparities (Supplementary Table S1).

A monitoring system could distinguish between GMPs with and without local bias, and could monitor the time trend of country level inequality. At present, there is no monitoring system, to inform stakeholders neither at local nor at the country level. Consequently, there is a pressing need for monitoring systems specific to SAs. Our investigation demonstrates not only the feasibility of segregation oriented monitoring but also suggests indicators for this purpose. However, it is evident that additional indicators are required to understand the processes leading to the observed disparities in premature mortality, health care use, and insurance reimbursement. This monitoring system could support the National Social Inclusion Strategy of the Hungarian government (29), and as Roma are overrepresented in SAs, it could contribute to the programs aimed at improving the health status of the Roma population. The effectiveness of interventions could be enhanced by considering self-declared Roma ethnicity (available from census data) in defining SAs.

## 5 Conclusion

This analysis showed that (1) in the Hungarian health system, there are varying degrees of GMP-level dissimilarity in both health services delivery and reimbursement, in addition to varying health status between people living in SAs and CAs; this suggests that residence in an SA is a strong factor impacting the health care services system. Furthermore, (2) some Hungarian GMPs seemed to provide equal care to their inhabitants, while others show varied levels of inequality. We suggest that further studies are required to investigate such variations and local factors affecting the quality of care provided to segregated populations. According to our findings (3) any decision-making on interventions related to SAs should take the local (GMP-level) environment into consideration.

## Data availability statement

The data analyzed in this study is subject to the following licenses/restrictions: the datasets used and/or analyzed during the current study are available from the corresponding author on reasonable request. Requests to access these datasets should be directed to janos.sandor@med.unideb.hu.

## Ethics statement

The protocol to produce segregation-specific indicators was approved by the Office of the Commissioner for Fundamental Rights (AJB-3147/2013), the general director of the NHIF (E0101/215-3/2014), and the Hungarian National Authority for Data Protection and Freedom of Information (NAIH/2015/826/7N). The studies were conducted in accordance with the local legislation and institutional requirements. Written informed consent for participation was not required from the participants or the participants’ legal guardians/next of kin in accordance with the national legislation and institutional requirements.

## Author contributions

FK prepared the literature review, analyzed, interpreted the data, and prepared the draft of the manuscript. FV prepared the primary database. LK helped with conception and data base preparation. RÁ helped with conception and study design. JS elaborated on the design, interpreted the results, and finalized the manuscript. All authors read and approved the final manuscript.
