# Supplementary material for: Cross-sectional comparison of health care delivery and reimbursement between segregated and nonsegregated communities in Hungary
Source: Front Public Health. 2024 Jan 24;12:1152555. doi: 10.3389/fpubh.2024.1152555 (PMC10847262; doi:10.3389/fpubh.2024.1152555)
Supplement: Supplementary file 1 [file Table_1.DOCX]

Supplementary Material

## Supplementary Table

| Indicators | GMPs with lower RR in SA than in CA | | | | GMPs with nondifferent RR in SA and CA | | | | GMPs with higher RR in SA than in CA | | |
| --- | --- | --- | --- | --- | --- | --- | --- | --- | --- | --- | --- |
|  | Number of GMPs* | Adult population in segregated areas | Excess of episodes among segregated adults | Number of GMPs* | | Adult population in segregated areas | Excess of episodes among segregated adults | Number of GMPs* | | Adult population in segregated areas | Excess of episodes among segregated adults |
| Healthcare delivery (episodes) | | | | | | | | | | | |
| GP visits | 1110 | 55137 | -71482.50 | 2078 | | 86538 | 1587.06 | 1164 | | 110626 | 165999.22 |
| Use of outpatient services without CT/MRI | 27 | 10026 | -1078.09 | 4262 | | 240184 | -4328 | 8 | | 2021 | 305.47 |
| Use of CT/MRI | 16 | 5111 | -169.62 | 2297 | | 227518 | 85.47 | 167 | | 6855 | 523.55 |
| Use of hospital service | 1 | 65 | -8.31 | 2757 | | 205279 | 3143.88 | 325 | | 40851 | 2931.05 |
| All cause premature mortality | | | | | | | | | | | |
| All cause premature mortality | 5 | 2759 | -73.78 | 558 | | 103334 | 137.39 | 125 | | 3616 | 158.83 |

- **Supplementary Table 1.** Number of GMPs, size of the adult population, and excess number of episodes of healthcare delivery and mortality among segregated patients compared to complementary patients. *Margins of error: GP visits (109), Outpatient service (107), CT/ MRI (62), hospital service (77), mortality (17).

.
